# Growth Abnormalities in Children with Chronic Hepatitis B or C

**DOI:** 10.1155/2012/670316

**Published:** 2012-03-04

**Authors:** P. Gerner, Andre Hörning, S. Kathemann, K. Willuweit, S. Wirth

**Affiliations:** ^1^Children's Hospital, University of Duisburg-Essen, Klinic 2, 45122 Essen, Germany; ^2^Children's Hospital, HELIOS Klinikum, 42283 Wuppertal, Germany

## Abstract

*Background*. It has been suggested that chronic hepatitis B infection leads to growth impairment, but data are inconsistent and underlying factors are not defined. *Methods*. Children and adolescents with chronic hepatitis B (HBV) or C (HCV) were retrospectively evaluated for growth, weight, antiviral treatment, biochemical signs of liver inflammation, route of infection, and HBV DNA, respectively. *Results*. In all, 135 children (mean age 6.1 years, 81 male, 54 female) with HBV (*n* = 78) or HCV (*n* = 57) were studied. Route of infection was vertical in 50%, parenteral in 11%, and unknown in 39%. ALT levels were above 1.5 times above normal in 30% while 70% had normal/near normal transaminases. 80% were Caucasian, 14% Asian, 1% black, and 4% unknown. Mean baseline height measured in SDS was significantly lower in the study population than in noninfected children (boys −1.2, girls −0.4, *P* < 0.01). 28 children were below 2 standard deviations of the norm while 5 were above 2 standard deviations. SDS measures in relation to individual factors were as follows: elevated ALT: boys −1.4, females −0.5 (*P* < 0.01), ALT normal/near normal: boys +0.4, females +0.6; parenteral transmission: boys −3.3, girls −0.9 (*P* < 0.01), vertical transmission: boys −0.2, females −0.2. Antiviral treatment itself or HBV-DNA load did not reach statistically significant differences. *Conclusions*. Chronic HBV or HCV may lead to compromised growth which is mostly influenced by liver inflammation. Our data may argue for early antiviral treatment in children with significant ALT elevation.

## 1. Introduction

It is estimated that more than half of the world's population has been infected with hepatitis B virus (HBV) or hepatitis C virus (HCV), and 400–550 million people are chronic carriers. Since both are not cholestatic liver diseases, neither chronic hepatitis B (CHB) nor chronic hepatitis C (CHC) have been associated with those liver disorders most likely to affect the nutritional status and growth of children. A number of potential causes of malnutrition in chronic liver diseases have been identified [1], but previous studies have suggested that children with chronic hepatitis B or C seem to be smaller than children without infection [2, 3]. However, relatively few studies with limited numbers of patients have evaluated the impact of CHB on children's growth [2–5]. Moreover, to our knowledge there is no study investigating growth of children with chronic hepatitis C. Furthermore, there is little knowledge about the influence of the specific virological status such as high or low viral load and elevated or normal transaminases, the route of infection and antiviral treatment.

To study the growth of children with CHB or CHC presents a number of challenges. Since growth itself is a periodic, saltatory, and pulsatile event, growth data are inherently noisy. The inherent noise in growth data is enhanced by the difficulty of obtaining accurate measurements in young children at regular intervals, which increases the number of children needed for the analysis to reach statistical significance. Moreover, if CHB or CHC does impact growth, it is reasonable to assume that the route of infection, and herewith the duration of infection, may be a factor in determining height at a given age. Unfortunately, the date of acquisition of a given infection is unknown in most cases except children who are perinatally infected. Finally, growth patterns vary widely among individuals, and there are notable differences in growth patterns between genders and races.

The aim of this study was to determine how CHB or CHC affects the growth of children. To accomplish this, we retrospectively monitored the growth and weight of 135 children and tried to identify factors influencing growth development.

## 2. Patients and Methods

Pediatric patients in this study are chronically infected with either hepatitis B or C. They were collected from three centers: Childrens' Hospital Essen University, Childrens' Hospital Helios Klinikum Wuppertal, and the Childrens' Hospital Klinikum im Friedrichhein Berlin. In a retrospective study all patients in these hospitals with the diagnosis of chronic HBV or HCV who were in medical attendance since the year 2000 were included in this study. The baseline characteristics of the 135 children included in this study are shown in Table 1.

The ethnic origin of our 135 patients is as follows: 63% are from Eastern Europe, 5% are of Asian origin, 1% are of African origin, 9% are of Western Europe, and 22% are of unknown origin. The 28 with significant growth retardation belong to all groups of these different regions.

Height and weight were measured in centimetres and kilograms, respectively. The anthropometric results of the last attendance in our hospital were chosen for each child.

Height and weight, as well as body mass index (BMI) percentiles, were determined from the last visit of the patient. The height data were calculated as SDS for chronological age using the tables derived from the Dortmunder longitudinal growth study [6]. Growth was considered to be impaired if SDS was less than −1.0. Liver inflammation was appreciated by measuring ALT levels. Moreover, other clinical factors as antiviral treatment, route of infection, HBV DNA load, race, sex, and age were evaluated.

In cases of significant growth retardation, as in the mentioned children, our routine investigations include exclusion of intestinal malabsorption and exclusion of growth hormone deficiency.

The significance of intergroup differences was studied with SPSS 11.0 computer program by chi-squared test, Wilcoxon-signed rank and Spearman correlation analysis. A *P* value of <0.05 was considered significant.

## 3. Results

**Figure 1:**
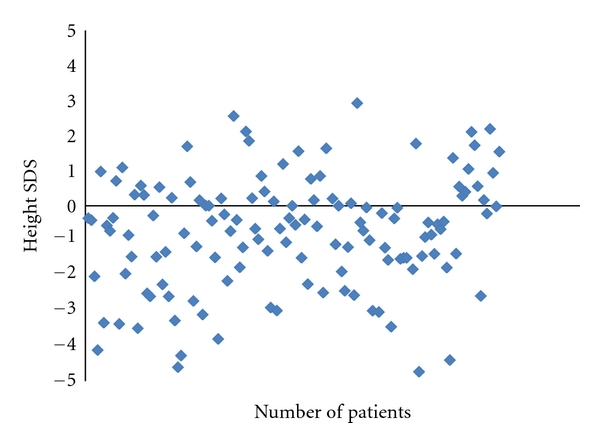
SDS from 135 children with chronic HBV or HCV infection. 43 children are above and 92 children are below SDS 0. Each dot represents one patient.

**Figure 2:**
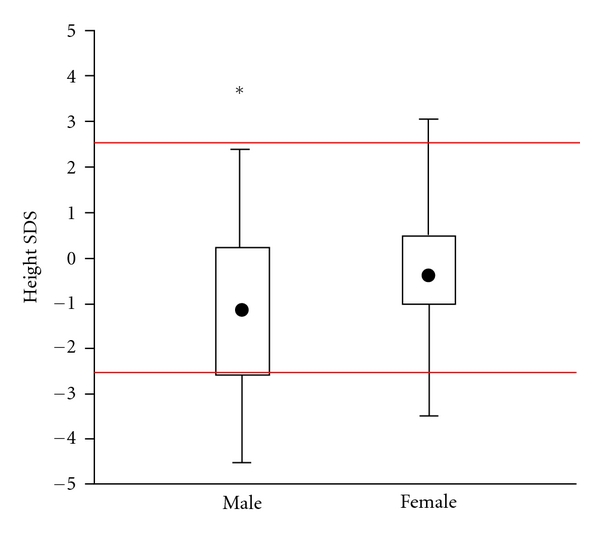
Comparison of SDS from 135 children with chronic HBV or HCV infection. *P < 0.01.

The SDSs for the 135 HBV- or HCV-infected children at their last visit in the three study centers are shown in Figure 1, the median SDS is shown in Figure 2. Since there was a difference between boys and girls, SDS is demonstrated for each group. The median SDS for both was −0.9 which corresponds to compromised height regarding a reference population as published [6]. The height of males tended to be more compromised than that of females; however, sex was only of borderline significance. 28 children showed significant growth impairment of more than 2 standard deviations (see Figure 1).

**Figure 3:**
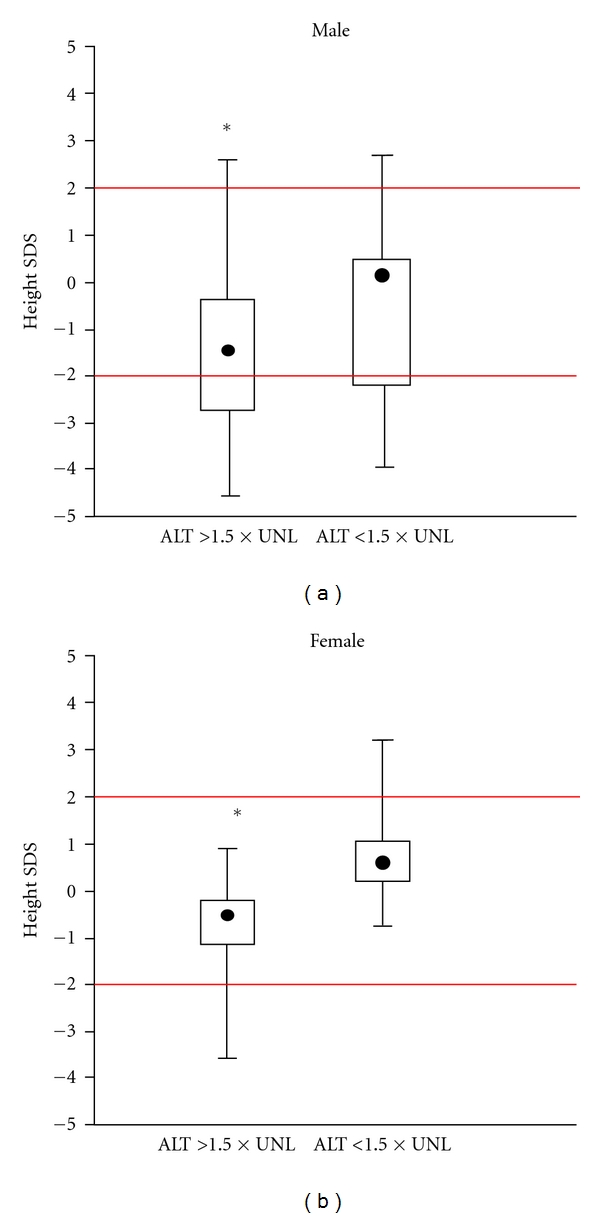
Comparison of SDS from 135 children with chronic HBV or HCV infection in relation to biochemical signs of liver inflammation measured by ALT. *P < 0.01.

**Figure 4:**
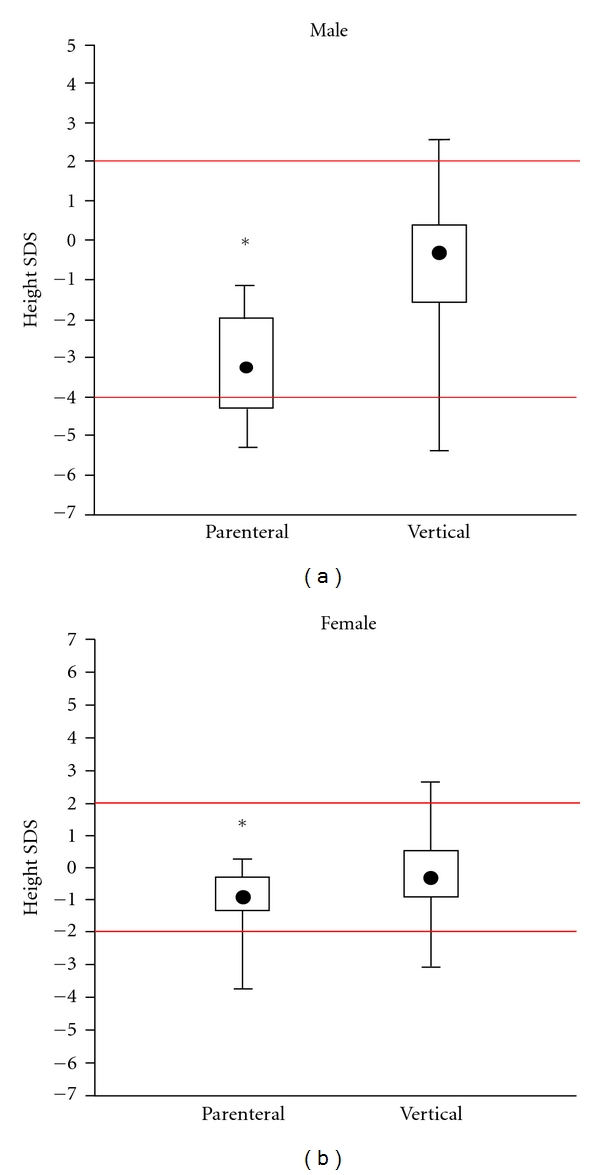
Comparison of SDS from 135 children with chronic HBV or HCV infection in relation to route of transmission. *P < 0.01.

**Figure 5:**
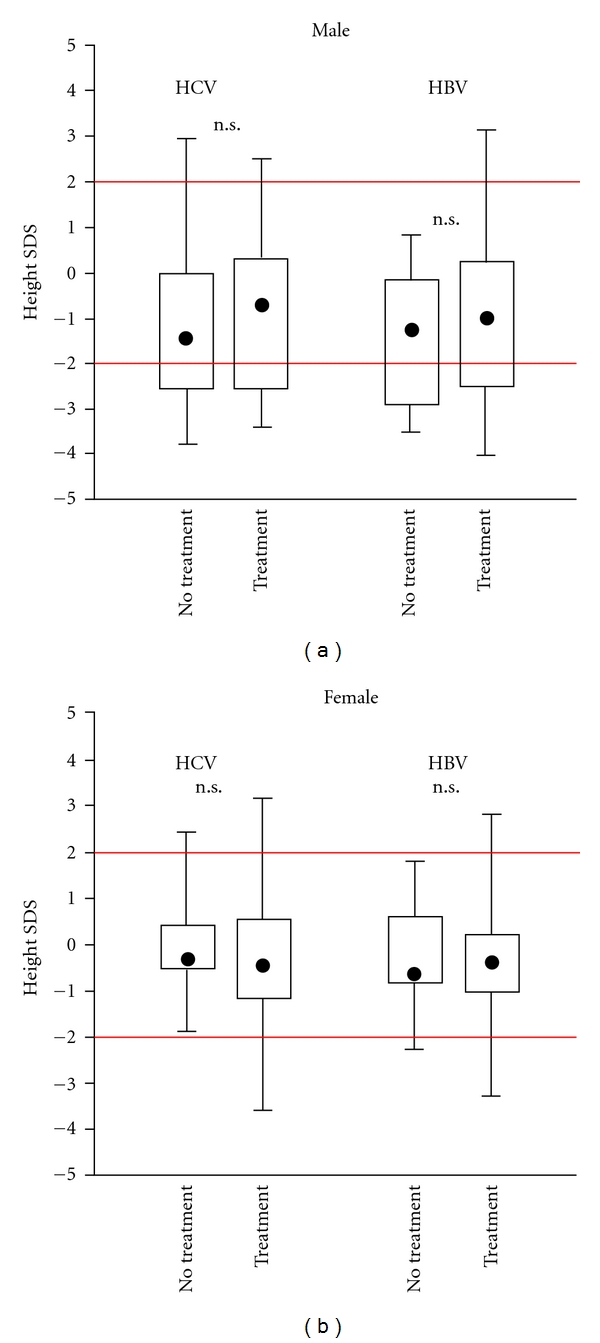
Comparison of SDS from 135 children with chronic HBV or HCV infection in relation to treatment. Treatment HCV: interferon Alpha monotherapy or in combination with ribavirin. Treatment HBV: interferon alpha or lamivudine. n.s., not significant.

**Figure 6:**
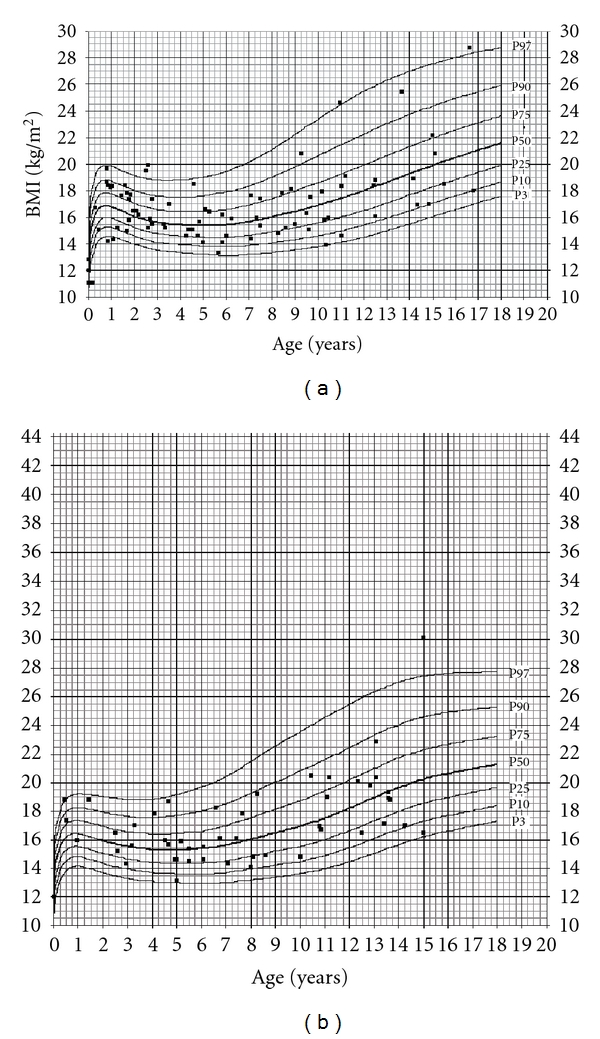
(a) Percentiles of BMI for boys. (b) Percentiles of BMI for girls.

The most significant difference from the reference population was found after comparing children with normal or near normal transaminases and those with ALT elevation. In Figure 3, SDS from children with significant ALT elevation is −1.4 and in those with near normal ALT +0.4 in males and −0.6 and +0.4 for females, respectively. A similar effect was found after comparing route of infection (Figure 4): children with parenteral infection were significantly smaller than those after vertical transmission. We also studied the influence of HBV DNA load from the 78 HBV-infected children, but there was no significant influence (data not shown). Moreover, prior antiviral treatment did not significantly show any difference (Figure 5). In HBV-infected children, there was only a tendency that children after treatment with alpha interferon or lamivudine were slightly taller than those without treatment. There was no difference in BMI as only 2 boys, and no girls were below the 3th percentile (Figures 6(a) and 6(b)).

## 4. Discussion

In this study, we evaluated the impact of chronic hepatitis B or C on growth of children and adolescents. We found that children with HBV or HCV showed compromised growth, and correlation with clinical variables was able to identify factors that might play a crucial role for growth abnormalities. In general, high transaminases were associated with short stature. The second factor that negatively influenced growth was parenteral transmission. These two factors are likely to be linked as children who are infected later in life (which is usually the case in parenterally transmitted individuals) are less likely to be in the so-called immunotolerant phase. This phase is a well-described phenomenon in childhood, and the great majority of children in this phase are perinatally (vertically) infected. As their immune system is not yet fully developed, T cells do less harm to infected hepatocytes by cytolytic processes. Therefore, in these children transaminases are within normal range for years or even decades until the immunotolerance against HBV switches to the immune competent phase. In our opinion, it is reasonable that the route of infection and liver inflammation reflect the same underlying factor for growth impairment. However, it has to be noted that in this study only 15 patients were transmitted parenterally, and therefore these data need to be confirmed in other trials. Our data are in line with another study consisting of 72 children with significant ALT elevation who were also found to be smaller than others [3]. In contrast, there is one report about Turkish pediatric patients in which no negative effect of hepatitis B on growth was demonstrated [7]. Due to the low number of only 34 immunoactive and 15 immunotolerant HBV patients involved, this study may have been underpowered. The finding of a negative effect of ALT elevation is surprising since liver biopsies of these children do rarely show significant liver damage [8]. Usually the liver shows no or only mild inflammation. Nevertheless, it seems possible that also mild but chronic liver inflammation is capable to lead to impaired growth. This explanation is only speculative and it is also possible, as with other chronic diseases, that the mechanism underlying compromised growth is multiple and complex. For example, factors such as decreased caloric intake, malabsorption of nutrients, effects of chronic liver disease on IGF production in the liver or inflammatory mediators may contribute to compromised growth in children with HBV or HCV. However, if verified in other studies our data provide one further argument for early antiviral treatment in children with biochemical signs of liver inflammation despite histologic mild pathology.

We also searched for the role of HBV itself. According to our data, the viral load did not influence growth. In several other studies with children and chronic viral infection, this was an important factor: in children with HIV [9], and asymptomatic CMV [10].

In summary, we found that the average child with chronic hepatitis B or C shows compromised growth. Further, we found that one important factor for growth impairment is ALT elevations. It is possible that treating HBV- or HCV-infected children with antiviral drugs may improve their growth in the long term. Which antiviral treatment (alpha-interferon or nucleos(t)ide analogues) is the best for long term development of the child still has to be determined.

## Figures and Tables

**Table 1:** Baseline characteristics of children infected with hepatitis B or C.

Age (years.)	
Median	6.1
Range	0–17

Height (cm)	
Median	118.2
Range	50–187.3

Weight (kg)	
Median	21
Range	3.1–78.4

Sex (*n*)	
Male	81
Female	54

AST (*n*)	
Elevated >2 × UNL	41
<2 × UNL	94

Route of Infection	
Vertical	68
Parenteral	15
Unknown	52

Race	
Caucasien	108
Black	2
Asien	19
Other	6
chronic Hepatitis B	*n* = 78

HBV-DNA (*n*)	
Low*	16
High	60
Not determined	2

Treatment HBV (*n*)	
Interferon	7
Lamivudine	14
Interferon, Lamivudine	6
None	51
chronic Hepatitis C	*n* = 57

Treatment HCV (*n*)	
Interferon + Ribavirin	35
None	22

*HBV DNA < 100.000 IU/mL.

## References

[1] Novy MA, Schwarz KB (1997). Nutritional considerations and management of the child with liver disease. *Nutrition*.

[2] Sokol RJ, Stall C (1990). Anthropometric evaluation of children with chronic liver disease. *American Journal of Clinical Nutrition*.

[3] Comanor L, Minor J, Conjeevaram HS (2001). Impact of chronic hepatitis B and interferon-alpha therapy on growth of children. *Journal of Viral Hepatitis*.

[4] Vegnente A, Guida S, Di Costanzo C (1992). Nutritional status and growth in children with chronic hepatitis B. *Journal of Pediatric Gastroenterology and Nutrition*.

[5] Polito C, La Manna A, Cartiglia ML (1991). Normal growth of children with HBsAg positive chronic active hepatitis (CAH). *Acta Paediatrica Scandinavica*.

[6] Reinken J, van Oost G (1992). Longitudinale Körperentwicklung gesunder Kinder von 0–18 Jahren. *Klinische Padiatrie*.

[7] Kuloğlu Z, Kansu A, Demirçeken F (2007). The influence of interferon-*α* and combination interferon-*α* and lamivudine therapy on height and weight in children with chronic hepatitis B infection. *Journal of Pediatric Endocrinology and Metabolism*.

[8] Guido M, Bortolotti F (2008). Chronic viral hepatitis in children: any role for the pathologist?. *Gut*.

[9] Hilgartner MW, Donfield SM, Lynn HS (2001). The effect of plasma human immunodeficiency virus RNA and CD4^+^ T lymphocytes on growth measurements of hemophilic boys and adolescents. *Pediatrics*.

[10] Zhang XW, Li F, Yu XW, Shi XW, Shi J, Zhang JP (2007). Physical and intellectual development in children with asymptomatic congenital cytomegalovirus infection: a longitudinal cohort study in Qinba mountain area, China. *Journal of Clinical Virology*.

